# Topical Drug Delivery of Concentrated Cabazitaxel in an α‐Tocopherol and DMSO Solution

**DOI:** 10.1002/advs.202302658

**Published:** 2023-08-09

**Authors:** Boyang Sun, Georgios Paraskevopoulos, Jiwei Min, Robert Rossdeutcher, Sanjana Ghosh, Breandan Quinn, Meng‐Hsuan Lin, Debanjan Sarkar, Dinesh Sukumaran, Yuefei Wang, Kateřina Vávrová, Jonathan F. Lovell, Yumiao Zhang

**Affiliations:** ^1^ School of Chemical Engineering and Technology Key Laboratory of Systems Bioengineering (Ministry of Education) Frontiers Science Center for Synthetic Biology (Ministry of Education) State Key Laboratory of Chemical Engineering Tianjin University Tianjin 300350 P. R. China; ^2^ Skin Barrier Research Group Faculty of Pharmacy Charles University Akademika Heyrovského 1203 Hradec Králové 50005 Czech Republic; ^3^ Department of Chemistry State University of New York at Buffalo Buffalo NY 14260 USA; ^4^ Department of Biomedical Engineering State University of New York at Buffalo Buffalo NY 14260 USA

**Keywords:** cabazitaxel, skin cancer, transdermal delivery, α‐tocopherol

## Abstract

Topical chemotherapy approaches are relevant for certain skin cancer treatments. This study observes that cabazitaxel (CTX), a broad‐spectrum second‐generation taxane cytotoxic agent, can be dissolved in α‐tocopherol at high concentrations exceeding 100 mg mL^−1^. 2D nuclear magnetic resonance (NMR) analysis and molecular dynamics (MD) are used to study this phenomenon. The addition of 30% dimethyl sulfoxide (DMSO) to the α‐tocopherol/CTX solution improves its working viscosity and enhances CTX permeation through human skin in vitro (over 5 µg cm^−2^ within 24 h), while no detectable drug permeates when CTX is dissolved in α‐tocopherol alone. In a transepidermal water loss assay, the barrier impairment induced by CTX in 30% DMSO in α‐tocopherol, but not in pure DMSO, is reversible 8 h after the formulation removal from the skin surface. Antitumor efficacy of the topical CTX formulation is evaluated in nude mice bearing A431 human squamous carcinoma skin cancer xenografts. With topical application of concentrated CTX solutions (75 mg mL^−1^), tumor growth is significantly suppressed compared to lower concentration groups (0, 25, or 50 mg mL^−1^ CTX). Taken together, these findings show that topical delivery of CTX using a DMSO and α‐tocopherol solvent warrants further study as a treatment for skin malignancies.

## Graphical abstract

Cabazitaxel (CTX) is a potent second generation taxane cytotoxic agent, soluble in α‐tocopherol at concentrations over 100 mg mL^−1^. In vitro tests on human skin show enhanced CTX penetration with DMSO. Mice treated topically with 75 mg mL^−1^ CTX in 30% DMSO in α‐tocopherol display effective antitumor results. This study opens a promising path for taxanes in topical skin cancer treatment.

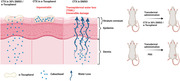

## 1 Introduction

Skin cancer is one of the most common human malignancies worldwide, and the non‐melanoma skin cancer (NMSC) subtype is estimated to have over 3 million newly diagnosed cases each year in the United States alone.^[^ ^1^ ^]^ Basal cell carcinoma (BCC) and squamous cell carcinoma (SCC) account for ≈95% of non‐melanoma skin cancer (NMSC) cases.^[^ ^2^ ^]^ Although the etiology of skin cancer is multifaceted, ultraviolet radiation of natural sunlight is recognized as the main risk factor.^[^ ^3^ ^]^ Photoproducts generated from ultraviolet radiation post oxidative damage to DNA cause characteristic mutations, which in turn lead to skin cancer.^[^ ^4^ ^]^ Approved by the United States Food and Drug Administration, 5‐Fluorouracil (5‐FU) is a standard treatment for actinic keratosis and NMSC.^[^ ^5^ ^]^ However, it has a series of drawbacks in clinical reports, including a strong inflammatory reactions, ≈50% ineffective treatment responses, and up to 55% of patients have observed recurrences,^[^ ^6^ ^]^ providing impetus for the development of additional topical skin cancer treatment options.

Taxanes, including paclitaxel (PTX), docetaxel (DTX), and cabazitaxel (CTX) are efficacious chemotherapy drugs that are used for systemic chemotherapy for the treatment of breast, lung, ovarian, and other malignant tumors.^[^ ^7^ ^]^ Owing to their ability to stabilize cell microtubule polymerization, taxanes prevent cell mitosis leading to apoptosis.^[^ ^8^ ^]^ As a second‐generation taxane drug, CTX was designed to overcome multi‐drug resistance owing to a low affinity to P‐glycoprotein.^[^ ^9^ ^]^ In a phase III clinical trial, CTX and prednisone treatment improved overall survival compared to mitoxantrone and prednisone treatment in metastatic, castration‐resistant prostate cancer that failed docetaxel‐based therapy.^[^ ^10^ ^]^ CTX has been assessed in many delivery formulations, including micelles,^[^ ^11^ ^]^ liposomes,^[^ ^12^ ^]^ lipid particles,^[^ ^13^ ^]^ serum albumin particles,^[^ ^14^ ^]^ polymeric nanoparticles,^[^ ^15^ ^]^ and covalent conjugates,^[^ ^16^ ^]^ However, to the best of our knowledge, CTX has not yet been explored for topical delivery.^[^ ^9^ ^]^

Transdermal administration of chemotherapy drugs is a non‐invasive, convenient, controlled‐release approach for skin cancer treatment.^[^ ^17^ ^]^ However, the natural stratum corneum skin barrier hinders drugs such as taxanes from penetrating the skin, so penetration improvements are needed.^[^ ^18^ ^]^ To enhance the skin penetration of docetaxel, Qiu et al. used a combination of microneedle and elastic liposomes, and found the lag time was decreased by ≈70% compared with conventional liposomes.^[^ ^19^ ^]^ DMSO is a safe, effective, and well‐known penetration enhancer, which has been used to promote hydrophilic and lipophilic drugs through the skin.^[^ ^20^ ^]^ Since 1978, many pharmaceutical preparations using high DMSO concentrations have been approved in the United States and European Union including Viadur for prostate cancer, Dolicur for interstitial cystitis and Pennsaid for knee osteoarthritis, and Herpid for herpes zoster.^[^ ^21^ ^]^ α‐tocopherol is an essential antioxidant compound found in skin tissues and has been widely used in cosmetics and clinical dermatology.^[^ ^22^ ^]^ Studies have shown that tocopherol succinate, an α‐tocopherol derivative has antitumor effects.^[^ ^23^ ^]^ The anticancer activity of tocopherol succinate comes from α‐tocopherol moiety and not the succinate salt.^[^ ^24^ ^]^ α‐tocopherol has also been shown to accelerate wound healing.^[^ ^25^ ^]^

**Figure 1:**
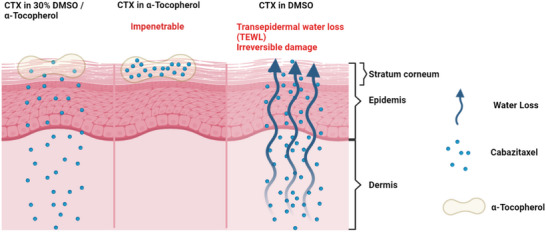
Schematic illustration of the therapeutic effect of CTX in α‐tocopherol, CTX in 30% DMSO/α‐tocopherol and CTX in DMSO, respectively. CTX in α‐tocopherol is unable to penetrate the skin layer while CTX in DMSO causes the transepidemal water loss (TEWL) in the skin.

In this work, to develop a topical formulation to treat non‐melanoma skin cancer, the potent anticancer drug CTX was found to be dissolvable in α‐tocopherol at a high concentration due to drug and solvent molecular interaction (**Figure** **1**). DMSO was added to adjust the viscosity of the formulation and to act as a skin penetration enhancer. The penetration kinetics and distribution of CTX in human skin in vitro showed efficient CTX delivery through the skin using 30% DMSO in α‐tocopherol. In vivo antitumor efficacy of CTX in 30% DMSO in α‐tocopherol was also evaluated in mice bearing subcutaneous A431 squamous cell carcinoma xenografts.

## 2 Results and Discussion

**Figure 2:**
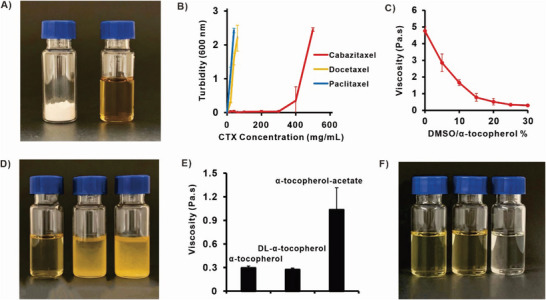
CTX can be dissolved in α‐tocopherol (Vitamin E) at high concentrations and solution viscosity can be adjusted with DMSO. A) Photograph of 100 mg of CTX powder (left) and 100 mg CTX powder dissolved in 1 mL α‐tocopherol (right). B) The turbidity (optical absorbance at 600 nm) of taxanes (CTX, DTX, PTX) dissolved in α‐tocopherol. C) The viscosity α‐tocopherol with varying amounts of DMSO added. D) Photograph of 100 mg of CTX (left) DTX (middle) PTX (right) in 30% DMSO/ α‐tocopherol. E) Viscosity or F) photographs of 100 mg CTX dissolved in 30% DMSO/α‐tocopherol, 30% DMSO/ DL‐α‐tocopherol, 30% DMSO/ α‐tocopherol‐Acetate. Data shows mean ± S.D. for *n* = 3.

The chemical structure of CTX is shown in Figure S4 (Supporting Information). With an octanal‐water partition coefficient (log P) of 3.9, CTX is nearly insoluble in water.^[^ ^26^ ^]^ In the course of experiments, we observed that CTX, a white powder, could be dissolved in α‐tocopherol (the most prevalent isoform of vitamin E) at extremely high concentrations (**Figure** **2**A). The solubility was remarkable, as CTX could dissolve at concentrations as high as 300 mg mL^−1^ (Figure 2B). Notably, paclitaxel and docetaxel did not share this high solubility in α‐tocopherol. CTX is a potent anticancer drug; hence, to avoid local toxicity while maintaining solubility, 100 mg mL^−1^ was used in most subsequent studies. This is an intermediary concentration range relative to other topical chemotherapy formulations such as Levulan (20% ALA) and Effudex (5% 5‐fluorouracil).

As α‐tocopherol is a highly viscous fluid, this could complicate topical application and also hinder drug diffusion and interaction with the skin. With the addition of DMSO, the rheological viscosity decreased (Figure 2C). CTX maintained good solubility when 30% DMSO was included in α‐tocopherol (v/v) (Figure 2D), while paclitaxel and docetaxel were not dissolved even with the help of DMSO. The viscosity of 30% DMSO in DL‐α‐tocopherol was comparable with that of 30% DMSO in α‐tocopherol, while 30% DMSO in α‐tocopherol acetate was more viscous (Figure 2E). DL‐α‐Tocopherol and α‐tocopherol acetate, each with 30% DMSO, also dissolved CTX at 100 mg mL^−1^ obtaining clear solutions, comparably to α‐tocopherol (Figure 2F).

**Figure 3:**
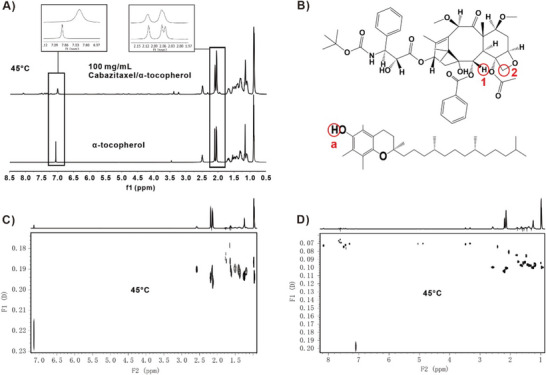
2‐D DOSY Nuclear Magnetic Resonance analysis of CTX/α‐tocopherol interaction. A) Stacked 1H‐NMR spectra of α‐tocopherol (bottom) and 100 mg mL^−1^ CTX/ α‐tocopherol (top) in 30% DMSO‐*d*_6_ at 45 °C. B) Chemical structure of CTX and α‐tocopherol. C) 2D‐DOSY spectra of α‐tocopherol in 30% DMSO‐*d*_6_ (500 MHz, 45 °C). D) 2D‐DOSY spectra of 100 mg mL^−1^ CTX / α‐tocopherol in 30% DMSO‐*d*_6_ (500 MHz, 45 °C).

Nuclear Magnetic Resonance was used to explore the high solubility of CTX in α‐tocopherol. The ^1^H‐NMR signals of α‐tocopherol were broadened in the presence of CTX at 35 and 45 °C (**Figure** **3**A; Figure S1A, Supporting Information). This indicates that α‐tocopherol forms an aggregated supramolecular entity in the presence of CTX that presumably accounts for the high solubility. Furthermore, the signals at ≈2.1 ppm showed a small downfield shift, while the one at ≈7.1 ppm showed a small upfield shift, indicating interactions of these protons in α‐tocopherol with the drug molecule.

To further understand the nature and extent of this possible supramolecular structure, we recorded DOSY spectra for α‐tocopherol with and without the drug at the same two temperatures (35 and 45 °C). DOSY measures the diffusion coefficients of molecules in solution and can predict molecular size in solution: larger molecular size leads to slower diffusion and lower diffusion coefficients. DOSY data presented in Figure 3C,D and Figure S1B,C (Supporting Information) showed lower diffusion coefficient values for α‐tocopherol in the presence of the drug at both temperatures. In addition, α‐tocopherol DOSY peaks show some distribution (of diffusion coefficients) while the CTX/ α‐tocopherol DOSY peaks were more compact, confirming a tightly organized structure of α‐tocopherol molecules in the presence of the drug. The obtained data indicate the presence of an aggregated structure of α‐tocopherol with CTX in solution. An empirical calculation of the molecular mass ratios from DOSY diffusion constants of α‐tocopherol in the presence and absence of the drug shows an aggregation factor of ≈7 and 8 in the presence of the drug. This indicates that one CTX molecule could have been encapsulated/solvated in an organized structure of ≈7 and 8 α‐tocopherol molecules. This is a plausible arrangement with the CTX polar groups interacting with α‐tocopherol phenolic hydroxyls and the tocopherol hydrocarbon moieties pointing in the opposite direction. Thus, α‐tocopherol appears to actively solvate CTX in solution.

The proximity of α‐tocopherol and the CTX molecules in the aforementioned arrangement was explored with 1H‐2D NOESY, which detects interactions between atoms that are <5 Å apart. Figure S1 (Supporting Information) shows NOESY correlations (presence of cross peaks) between the proton at 7.1 ppm (labeled a) in α‐tocopherol and the protons ≈5.85 ppm (labeled 1) and 4.5 ppm (labeled 2) in CTX. Calculated NMR spectra using the prediction software ACD Spectrus Processor identified these protons as the phenolic and the methyl protons on the rings of α‐tocopherol respectively. Figure 3A,B shows NOESY correlation peaks between proton a in α‐tocopherol and protons 1 and 2 in the drug (shown in inset box). We also see some cross‐peaks between α‐tocopherol peak at 2.1 ppm and drug peaks as well. This observation supports our postulate that the aromatic heads of α‐tocopherol molecules solvate the drug molecule.

**Figure 4:**
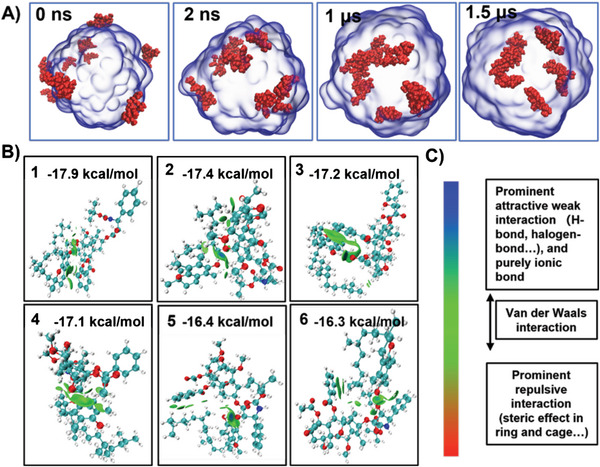
A) Molecular dynamics simulations of CTX dissolving in α‐tocopherol at 0, 2, 1000, and 1500 ns. The drug molecules (red) gradually dissolve into the α‐tocopherol solvent (blue). B) The six conformations in terms of free energy of binding are mostly green at the interaction interface. By combining the colorimetric card and the description in C) Intermolecular interactions are found to be mainly van der Waals interactions.

We further investigated the solubility of CTX in α‐tocopherol by Molecular Dynamics (MD) simulations. First, we built a model consisting of a sphere composed of α‐tocopherol solvent molecules and CTX drug molecules were randomly placed around the sphere. As shown in **Figure** **4**A, the model was placed in a square box and MD simulations of 1.5 µs duration were performed using GROMACS 2021.3.^[^ ^27^ ^]^ The drug molecules were found to be able to effectively move into the α‐tocopherol solvent sphere, suggesting that α‐tocopherol could solubilize CTX drug molecules (Video S1, Supporting Information). The RMSD (Root Mean Square Deviation) (Figure S5, Supporting Information) of the process converged at ≈1000 ns.

Since both drug and solvent molecules have a large number of atoms, we used MD simulations to compare the solubility of different drug molecules by calculating the free energy change (ΔG) when the drug molecule is added to the solvent.^[^ ^28^ ^]^ Here we compare two drug molecules, CTX and DTX (Figure S6A, Supporting Information). During the calculations, we linearly decoupled the van der Waals interactions between the drug molecule and the α‐tocopherol to obtain the free energy in different states, which allowed us to obtain ΔG during dissolution, as shown in Figure S6B,C (Supporting Information). The ΔG of the two molecules is very similar due to their similar structures, but overall, the ΔG of the CTX molecule is still 1 kcal mol^−1^ higher than that of the Docetaxel molecule (Figure S6C, Supporting Information).

In order to investigate the intermolecular interactions between CTX and α‐tocopherol in more detail, the Molclus program,^[^ ^29^ ^]^ was used to generate 200 dimers consisting of a drug molecule and an α‐tocopherol molecule, and they were used as initial models for structural optimization, and finally, single point energy calculations were performed using Gaussian 09,^[^ ^30^ ^]^ with high accuracy to obtain the 20 lowest energy conformations, and the six lowest energy configurations were subjected to IGMH,^[^ ^31^ ^]^ analysis using Multiwfn,^[^ ^32^ ^]^ as shown in Figure 4A,B and Figure S7A,B (Supporting Information), and the weak interactions were also decomposed under the molecular force field, as in Figure S7C (Supporting Information). The results showed that the weak interactions between CTX and α‐tocopherol were mainly van der Waals interactions, such as π–π stacking between benzene rings and p‐π stacking between benzene rings and alkyl chains, while electrostatic interactions only accounted for a smaller proportion (Figure 4C).

**Figure 5:**
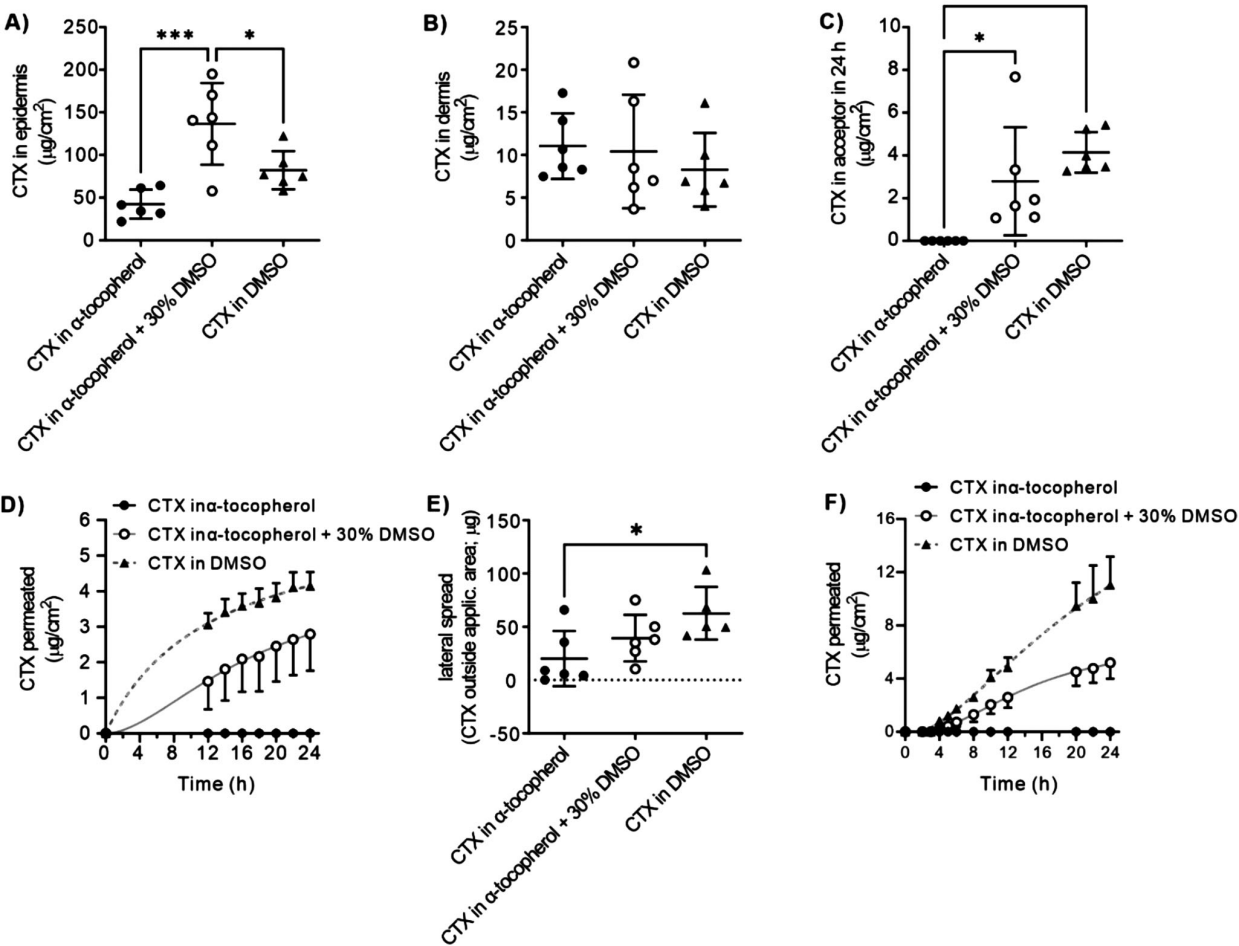
CTX permeation through human skin and retention in skin layers in vitro. CTX detected in A) human epidermis, B) dermis, and C) acceptor compartment after the application of 40 µL of 100 mg mL^−1^ CTX in α‐tocopherol, α‐tocopherol with 30% DMSO, and DMSO for 24 h (*n* = 6, * significant at *p* < 0.05, ***p < 0.001). (40 µL of the formulation applied on 1 cm^2^ skin); D) CTX permeation kinetics profiles from α‐tocopherol (*n* = 3), α‐tocopherol with 30% DMSO (*n* = 5), and DMSO (*n* = 6, means and S.D.). E) Cumulative amount of CTX that permeated through the skin in 24 h. F) CTX permeation profiles from a second permeation experiment with different skin donor and sampling intervals.

The CTX permeability through human skin and its retention in skin layers were evaluated in vitro by using Franz diffusion cells with a permeation area of 1 cm^2^. The CTX amounts in the epidermis were 42.5 ± 16.9, 136.5 ± 47.9, and 82.3 ± 16.9 µg cm^−2^ after 24 h application of 10% CTX formulations in α‐tocopherol, 30% DMSO in α‐tocopherol, and DMSO, respectively (**Figure** **5**A). The CTX amounts that reached the dermis were ≈10 µg cm^−2^ regardless of the formulation (Figure 5B). When applied in α‐tocopherol, no CTX was detected underneath the skin in the acceptor compartment. As the Figure 5D shows, the partial or full replacement of α‐tocopherol with DMSO significantly increased the cumulative amount of CTX that permeated through the skin in 24 h to 2.8 ± 2.5 µg cm^−2^ (30% DMSO) and 4.1 ± 1.0 µg cm^−2^ (100% DMSO; Figure 5C,D). In agreement with its effect on CTX permeation into the acceptor phase, the addition of DMSO increased the CTX lateral spread outside the 1 cm^2^ application area (Figure 5E). Total CTX amounts recovered from the skin and acceptor were 74 ± 33, 189 ± 53, and 154 ± 41 µg, using CTX in α‐tocopherol, 30% DMSO in α‐tocopherol, and DMSO, respectively. Figure 5F shows the CTX permeation profiles from a second permeation experiment with different skin donors and earlier sampling intervals. The cumulative CTX amounts that permeated through the skin in 24 h were 0, 5.2 ± 2.7, 11.0 ± 5.2 µg when applied in α‐tocopherol, 30% DMSO in α‐tocopherol, and DMSO, respectively.

Although the CTX quantities delivered through the skin comprise ≈5% of the applied drug, these results are remarkable for taxane permeation into and through human skin. For example, tyrosine‐derived nanospheres enabled the delivery of paclitaxel into the human epidermis at concentrations >100 ng cm^−2^, nanocarriers containing protein transduction domains achieved ≈9 µg cm^−2^ paclitaxel in porcine epidermis and 0.5 µg cm^−2^ in the acceptor in 12 h, nanostructured lipid carriers delivered ≈20 and 6 µg cm^−2^ paclitaxel in the porcine stratum corneum and deeper skin, respectively,^[^ ^33^ ^]^ Thus, 30% DMSO/α‐tocopherol solution represents an attractive option for treating skin tumors that spread into and below epidermal layers, as this formulation delivered more CTX into and through the skin than α‐tocopherol or DMSO alone. CTX in α‐tocopherol could be an interesting treatment alternative for initial epidermis‐confined carcinomas with reduced systemic absorption.

**Figure 6:**
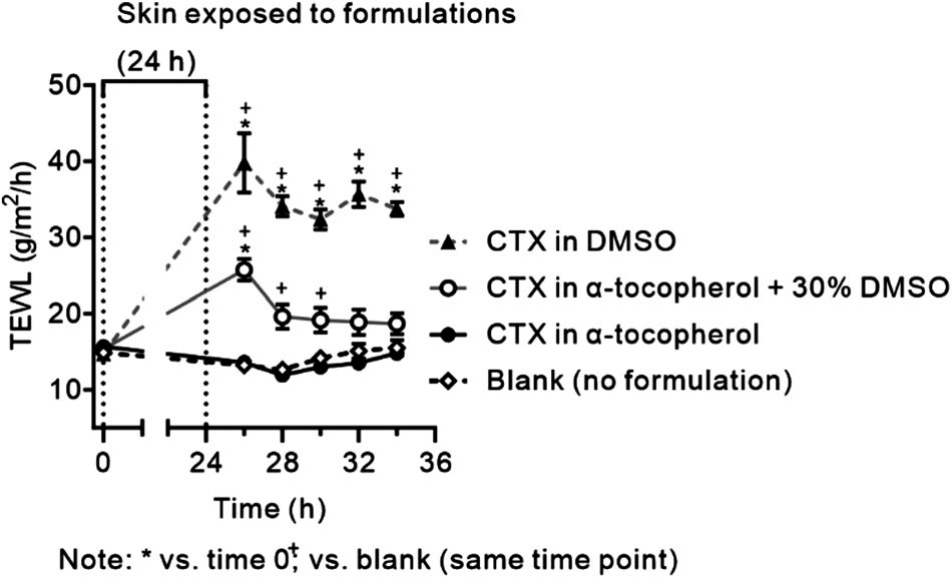
The effects of the formulations on TEWL and the irreversibility of that effect. Means and S.D., *p < 0.05 versus time 0; ^+^ *p* < 0.05 versus blank at the same time point; *n* = 5 (blank), *n* = 3 (α‐tocopherol), n = 5 (30% DMSO), *n* = 6 (DMSO).

The effects of topical or transdermal formulations on the skin barrier should be temporary; the barrier function should quickly recover after removing the formulation from the skin surface. Transepidermal water loss (TEWL) is a common dermatological measure of the skin barrier properties and indicates stratum corneum functional status.^[^ ^34^ ^]^ The skin barrier function was not affected by the α‐tocopherol formulation but decreased with increasing DMSO in the sample, as indicated by TEWL recorded on human skin in vitro (**Figure** **6**A). Importantly, the barrier impairment induced by CTX in 30% DMSO in α‐tocopherol was reversible within 8 h after the formulation removal from the skin surface. In contrast, TEWL of the skin treated with CTX in neat DMSO remained elevated over the baseline within the studied 10 h. The CTX in α‐tocopherol did not have any adverse effect on TEWL.

The primary mechanism of action of CTX in 30% DMSO/α‐tocopherol, which involves stabilizing microtubule polymerization and arresting cell mitosis, was assessed using a porcine tubulin assay. Figure S8 (Supporting Information) illustrates the evaluation of various formulations at a concentration of 10 µm on microtubule polymerization. As expected, CTX in 30% DMSO/α‐tocopherol demonstrated a significant increase in the rate of tubulin polymerization compared to the standard paclitaxel. In contrast, the vehicle consisting of 30% DMSO/α‐tocopherol and 5‐FU did not exhibit any disruptive effects on tubulin, similar to the PBS control. These findings indicate that CTX in 30% DMSO/α‐tocopherol possesses a potent ability to enhance tubulin polymerization, suggesting its efficacy in interfering with microtubule dynamics and cell mitosis. This mechanism of action is distinct from that of 5‐FU and the vehicle alone.

**Figure 7:**
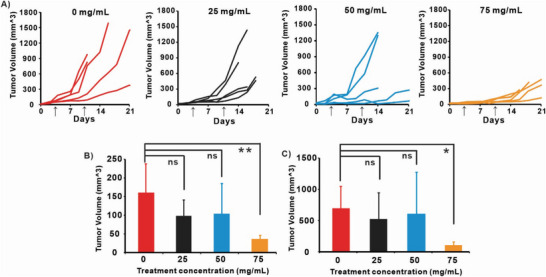
In vivo tumor inhibition efficacy on mice bearing subcutaneous A431 human skin cancer xenografts. A) Tumor volume change after received 20 µL of topical 0, 25, 50, and 75 mg mL^−1^ CTX in 30% DMSO/ α‐tocopherol on day 3 and 10. Arrow indicates drug administration dates. Statistically significant difference (ANOVA) was test for the tumors on B). day 7 and C). day 14 with ANOVA and Turkey's post‐test (*p < 0.5, **p < 0.05.).

The antitumor activity of topical CTX in 30% DMSO/α‐tocopherol was evaluated on female nude mice that were inoculated A431 skin tumor under their back‐skin pad. On day 3 and 10, 20 µL of 0, 25, 50, and 75 mg mL^−1^ CTX in 30% DMSO/ α‐Tocopherol formulations were smeared on the mouse tumor area, and individual tumor volume was measured as **Figure** **7**A shows. At high concentrations (75 mg mL^−1^), the tumor growth was delayed or suppressed while at low concentration (25  and 50 mg mL^−1^) the tumors were hardly inhibited compared with control group (0 mg mL^−1^). On day 7, compared with the 0 mg mL^−1^ group and the 75 mg mL^−1^ group had significant tumor suppression (Figure 7B). On day 14, the 75 mg mL^−1^ group still had a significant therapeutic effect compared with the control group (Figure 7C). It should be noted that the controls with CTX dissolved in DMSO alone and α‐tocopherol were not included due to the irreversible water loss induced by DMSO alone (Figure 6), and the high viscosity of α‐tocopherol alone causing it to be difficult to work with, as well as CTX being unable to penetrate the skin (Figure 5C,D).

**Figure 8:**
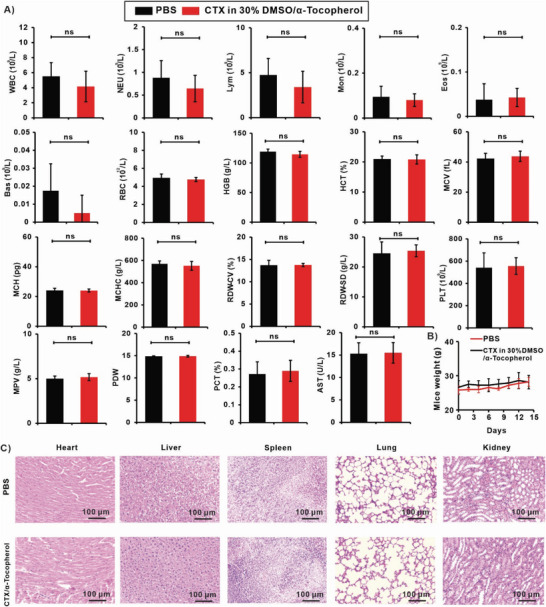
Systemic toxicity analysis of CTX formulations: CD‐1 Mice received transdermal administration of 20 µL 75 mg mL^−1^ CTX in 30% DMSO/α‐tocopherol at day 0 and 7 for toxicity analysis experiments (*n* = 5), at day 14 mice were sacrificed for toxicity analysis. A) Mice blood routine blood test and serum chemistry profile. B) Mice weight was monitored and recorded for 14 days. C) The H&E staining analysis of major organs. Statistical significance was analyzed via one‐way ANOVA with Tukey's post‐test: ns *p* > 0.05.

To evaluate the potential toxicity of CTX transdermal formulations, an in vivo study was conducted using outbred CD‐1 mice aged 6–8 weeks. The mice were divided into two groups, with the treated group receiving transdermal administration of 20 µL of 75 mg mL^−1^ CTX in 30% DMSO/α‐tocopherol. After a 14‐day treatment period, blood and organs were collected for comprehensive analysis, including complete blood count (CBC), serum chemistry profile, and histological hematoxylin and eosin (H&E) analysis (**Figure** **8**A). CBC parameters, including white blood cell (WBC) count, red blood cell (RBC) count, lymphocytes (LYM), mean cell volume (MCV), and others, showed no significant differences compared with the control group mice. Furthermore, H&E staining analysis of major organs did not reveal any signs of inflammation or other abnormalities (Figure 8C). Mouse weight also showed no changes (Figure 8B), suggesting that the transdermal formulation of CTX in 30% DMSO/α‐tocopherol did not induce overt acute toxicity in the mice. These results indicate the systemic safety of the CTX transdermal formulation, at least in this murine model with this dosing. Potential inflammatory reaction was also assessed by measuring serum levels of inflammatory cytokines using CD‐1 mice following transdermal administration of CTX formulation or PBS. Mice were topically administered with 20 µL of 75 mg mL^−1^ CTX in 30% DMSO/α‐tocopherol, while mice in the control group received PBS. After a 3‐day period, sera were collected and the expression of IL‐6, IL‐1β, IFN‐γ, and TNF‐α was measured using ELISA kits. Preliminary tests showed that after topical CTX treatment, there was a slight elevation in serum IFN‐γ, while levels of IL‐6, IL‐1β, and TNF‐α were not different from control, indicating that CTX in 30% DMSO/α‐tocopherol, when administered transdermally, does not elicit a strong inflammatory response (Figure S9, Supporting Information). The slight increase in IFN‐γ might be associated with promoting antitumor immune responses that activate the immune responses and induce immunogenic cell death (ICD) in tumors for the enhancement of the cancer treatment efficacy.^[^ ^35^ ^]^

## 3 Conclusion

To the best of our knowledge, there have been no reports on transdermal administration of CTX for skin cancer treatment, and in general topical formulations of taxanes are not frequently reported. Herein, we explored a novel topical chemotherapy formulation based on the high solubility of CTX in α‐tocopherol for skin malignancies. 2D NMR  and molecular dynamics simulations were employed to account for the high solubility at a molecular level. In vitro drug permeability tests conducted on human skin confirmed that DMSO enhanced CTX penetration. Without the addition of DMSO, no detectable CTX amounts penetrated through the skin after 24 h, while the 30% DMSO/α‐tocopherol mixture and DMSO alone efficiently delivered CTX. 30% DMSO in α‐tocopherol induced some water loss in TEWL test but it could be reversed within 8 h after the formulation was removed from the skin surface, unlike using pure DMSO as a carrier. In vivo antitumor tests in mice also showed a therapeutic effect using 75 mg mL^−1^ CTX in 30% DMSO in α‐tocopherol. In summary, a promising tropical CTX formulation has been developed by simply dissolving CTX in 30% DMSO/α‐tocopherol. Further studies are required to better study local drug delivery phenomenon with respect to pharmacokinetics, absorption, distribution, metabolism, and excretion, as well as local toxicity. Overall, this work offers a new potential avenue to explore the application of taxanes for topical skin cancer treatment.

## 4 Experimental Section

### Materials

DMSO was purchased from Fisher (# D128‐1). D‐α‐Tocopherol (# T2309), DL‐α‐Tocopherol (# T0251), and DL‐α‐Tocopherol Acetate (# T0252) were purchased from TCI. CTX was purchased from Carbosynth (# FC19621). Phosphate buffer saline (PBS) at pH 7.4 and solvents (HPLC grade) were purchased from Merck (Darmstadt, Germany). The aqueous solutions were prepared using ultrapure water (Milli‐Q RG water purification system, Millipore, Burlington, MA, USA). DMSO‐*d_6_* was purchased from Cambridge Isotope Laboratories, Inc.

### Formulation Preparations

To prepare the CTX formulation, the calculated amount of CTX powder was dissolved in DMSO, then added into α‐tocopherol or α‐tocopherol derivatives, and stirred until the formulation fully dissolved. For CTX, PTX, and DTX dissolved in α‐tocopherol, the calculated amount of drug was added to α‐tocopherol and sonicated until fully dissolved.

### Nuclear Magnetic Resonance

NMR studies were conducted on two samples – α‐tocopherol in 30% DMSO‐*d_6_* and α‐tocopherol plus the drug CTX in 30% DMSO‐*d_6_* (100 mg of drug per mL of Α‐Tocopherol). Spectra were recorded at two temperatures: 35 and 45 °C. All NMR spectra were collected on a Varian Inova Spectrometer operating at 500 MHz with a 5 mm PFG triple resonance probe. Spectra were obtained with standard pulse sequences provided by the vendor (sw = 4500 Hz, at = 3.6 s, d1 = 1 s, nt = 16). For this study, standard ^1^H‐1D, DOSY^1^ (Diffusion‐Ordered Spectroscopy BPPSTE), and ^1^H‐2D‐NOESY^2^ (Nuclear Overhauser Effect Spectroscopy) were collected for the samples described above. All spectra were processed using VNMRJ and MestReNova (v 11.0.4). Partial assignments of both α‐tocopherol and CTX were predicted using the prediction software ACD Spectrus Processor.

### Rheological Characterizations

The rheological properties of α‐tocopherol samples were characterized using a rheometer (Bohlin CVOD 100NF). All measurements were performed using a flat steel parallel plate geometry (10 mm) at 20 °C with a gap distance of 150 µm. The viscosity of the samples was analyzed in a controlled shear rate mode with a fixed shear rate of 10^−1^ s. All rheological experiments were performed three times for each sample, and the viscosity was plotted over the concentration (%) of DMSO in α‐tocopherol. All values were presented as average ± standard deviation (S.D.).

### Molecular Dynamics (MD) Simulation

To investigate the solubility of CTX in VE, molecular dynamics simulations of the system containing CTX and α‐tocopherol were carried out using GROMACS 2021.3,^[^ ^27^ ^]^ for 1.5 µs at a step size of 0.002 ps under NVT (isothermal‐isovolumetric) synthesis, using the Amber ff99SB‐ILDN force field,^[^ ^36^ ^]^ Energy minimization (1000.0 KJ mol^−1^ nm^−1^) was performed using the conjugate gradient method. The system was then pre‐equilibrated with isothermal‐isovolumetric (NVT, 2.0 ns, 298.15 K). Finally, a 1.5 µs production simulation at 298.15 K was carried out. The particle mesh Ewald (PME) method was used to deal with the system charges. The structure was placed in a rectangular box with a margin of 0.8 nm (i.e., the box has a side length of 5.8 nm). The initial model was constructed using Packmol.^[^ ^37^ ^]^The α‐tocopherol solvent spheres consist of 200 α‐tocopherol molecules with 10 CTX molecules randomly placed near the spheres. The topology of α‐tocopherol and CTX was generated by *acpype.py*,^[^ ^38^ ^]^ and the RESP charge was calculated by Gaussian 09,^[^ ^30^ ^]^ combined with Multiwfn,^[^ ^32^ ^]^ Gaussian 09 was first used to perform structural optimization of the α‐tocopherol and CTX, using the B3LYP theory method,^[^ ^39^ ^]^ and 6–31G* basis,^[^ ^40^ ^]^ and then to calculate the single point energies, using B3LYP theory method,^[^ ^39^ ^]^ and 6–311G** basis.^[^ ^41^ ^]^ DFT‐D3,^[^ ^42^ ^]^ dispersion correction had been added to all DFT calculations. The molecular structures and interactions were illustrated by visual molecular dynamics (VMD)^[^ ^43^ ^]^

### Free Energy Calculation

To investigate the solubility of different drugs (Cabazitaxel and Docetaxel) in VE, multiple 1 ns MD simulations were carried out using GROMACS 2021.3,^[^ ^27^ ^]^ for systems containing drug molecules and α‐tocopherol at NPT system synthesis with a step size of 0.001 ps, and the integrator was sd, using the Amber ff99SB‐ILDN,^[^ ^36^ ^]^ force field. Energy minimization (1000.0 kJ mol^−1^ nm^−1^) was performed using the steepest descendant algorithm. The system was then pre‐equilibrated with isothermal‐isovolumetric (NVT, 100 ps, 298.15 K) and isothermal‐isobaric (NPT, 100 ps, 298.15 K, 1 bar), respectively. Finally, the 1500 ns production simulation at 298.15 K was carried out with the Parrinello–Rahman barostat (1.0 bar). The particle mesh Ewald (PME) method was used to deal with the system charges. The system was placed in a cubic box with a side length of 6 nm and there was one drug molecule and 243 α‐Tocopherol molecules in it. Twenty‐one molecular dynamics simulations were performed for each drug molecule, each time with a different value of *λ* for van der Waals set in the MDP file. The margin of *λ* value between two adjacent simulations was 0.05, and only van der Waals interactions were considered during the simulations. The results of ΔG were obtained by applying the analysis command gmx_bar in GROMACS.

### Intermolecular Interactions Calculations

The software genmer in Molclus program,^[^ ^29^ ^]^ was applied to generate 200 dimers consisting of one drug molecule and one *α‐tocopherol* molecule. The 200 dimers were geometrically optimized using the semi‐empirical method of PM6‐DM+,^[^ ^44^ ^]^ in MOPAC2016,^[^ ^45^ ^]^ and the optimized structures were ranked in terms of energy using isostat in Molclus program.^[^ ^29^ ^]^ The 50 lowest energy structures were then optimized using the GFN2‐Xtb,^[^ ^46^ ^]^ method in the xtb,^[^ ^47^ ^]^ software, and the 20 lowest energy structures were then used to calculate the single point energy in Gaussian 09,^[^ ^30^ ^]^ using B3LYP theory method,^[^ ^39^ ^]^ and 6–311G** basis,^[^ ^41^ ^]^ The first six conformations were taken for IGMH,^[^ ^31^ ^]^ analysis using Multiwfn,^[^ ^32^ ^]^ to visualize the weak intermolecular interactions. The energy decomposition was also done under the molecular force field in Multiwfn.^[^ ^48^ ^]^

### Tubulin Polymerization

The kinetics of tubulin polymerization were assessed using the tubulin polymerization assay kit (BK006P, Cytoskeleton, Denver, CO, USA). Purified porcine brain tubulin was diluted with tubulin buffer to a concentration of 3 mg mL^−1^ and stored at −80 °C until further use. To measure the rate of tubulin polymerization, the tubulin solution was combined with 10% glycerol, 1 mm GTP, and 10 µm of the respective drug formulations. The mixture was preheated to 37 °C and rapidly transferred to a 96‐well plate reader to measure the absorbance at 340 nm every minute for a duration of 30 min at 37 °C.

### Evaluation of Inflammatory Cytokines in Mice

A total of 12 CD‐1 mice (6–8 weeks old) were randomly assigned to three groups, with each group consisting of four mice. The mice were topically administered 20 µL of CTX in a 30% α‐tocopherol/DMSO formulation. The control group received PBS. After a 3‐day period, the mice were sacrificed, and blood samples were collected. The serum obtained from the blood samples was then used for quantification using ELISA kits (Solarbio, Beijing, China).

### In Vivo Antitumor Efficacy and Toxicity Study

Animal studies were in compliance with the University at Buffalo IACUC protocols (BME04112Y). For tumor studies, cultured A431 (ATCC cat # CRL‐1555) skin cancer cells were injected subcutaneously in nude mice. After a three‐day period, 20 µL of the CTX formulations were topically applied onto the tumor area, and the drug formulation was administered as indicated until the completion of the antitumor efficacy study. For the toxicity study, healthy female CD‐1 mice were randomly divided into two groups (*n* = 5) and the hair on the dorsal region of each mouse was removed. On day 3 and 10, the treated group received transdermal administration of 20 µL of 75 mg mL^−1^ CTX in 30% DMSO/α‐tocopherol for toxicity analysis experiments. Mice weights were monitored for 14 days. On day 14, all mice were sacrificed and blood samples were collected via face piercing, suspended in citrate, and kept for further complete blood count (CBC) analysis and blood chemistry analysis. Organs including hearts, livers, spleens, lungs, and kidneys were harvested and fixed in formalin solution for subsequent hematoxylin and eosin (H&E) staining analysis.

### In Vitro Human Skin Permeation Experiments

Human skin from Caucasian females who underwent abdominal plastic surgery was used with the approval of the Ethics Committee of the Sanus Surgical Centre (4/5/2018), according to the principles of the Declaration of Helsinki. Informed consent had been obtained. The subcutaneous fat was carefully removed, and the remaining full‐thickness skin fragments were washed with saline, blotted dry, and stored at −20 °C.

CTX permeability through human skin and its retention in skin layers were evaluated in vitro using Franz diffusion cells with a permeation area of 1cm^2^ and acceptor volume of 7.0 ± 0.2 mL. Human skin was slowly thawed and dermatomed to a thickness of 0.6 mm with an Acculan 3TI dermatome (Aesculap, Center Valley, PA, USA). The dermatomed skin fragments were mounted into the cells with the dermal side facing the acceptor compartment, using Teflon holders sealed with silicone grease. Each acceptor compartment was filled with PBS at pH 7.4 with 25% ethanol and stirred at 32 °C throughout the experiment. Ethanol was added to ensure CTX solubility in the acceptor phase to maintain sink conditions as described before for docetaxel and paclitaxel.^[^ ^18^, ^19^ ^]^ Previously, no adverse effects of 25% ethanol in the acceptor phase on the skin permeability were observed. In a preliminary experiment, we compared CTX permeation and skin barrier function for 25% ethanol in PBS and 5% albumin in PBS as acceptor phases and found no significant differences.

After 6 h equilibration, the formulations (40 µL of 10% CTX in α‐Tocopherol, α‐Tocopherol with 30% DMSO, and DMSO) were evenly applied to the skin surface. Samples of the acceptor phase (0.3 mL) were withdrawn at predetermined time intervals up to 24 h and replaced with the same volume of the acceptor phase (25% EtOH in PBS). The amount of CTX permeated through the skin was analyzed by High‐Performance Liquid Chromatography (HPLC). The cumulative amount of CTX, corrected for the acceptor phase replacement, was plotted against time.

At 24 h, the formulations were carefully removed from the skin using water‐soaked cotton swabs. The cells were dismounted, and the skin was carefully washed again. The tissue exposed to the donor sample was punched out, wrapped in aluminum foil, and heated to 80°C for 1 min. The epidermis was then carefully peeled off from the dermis. The epidermis, dermis, and skin outside the application area were then weighed and extracted with 1, 1, and 2 mL methanol, respectively, for 24 h. The extracts were filtered and analyzed by HPLC. Mass balance experiments found that the amount of CTX recovered from the residual formulation, total skin, and acceptor phase was within 99 ± 19% of the applied amount.

### High‐Performance Liquid Chromatography (HPLC)

CTX was analyzed with a Shimadzu Prominence instrument (Shimadzu, Japan) consisting of LC‐20AD pumps with a DGU‐20A3 degasser, SIL‐20A HT autosampler, CTO‐20AC column oven, SPD‐M20A diode array detector, CBM‐20A communication module, and LCsolutions 1.22 software on an Agilent Eclipse XDB‐C18, 150 × 4.6 mm column with 5 µm particles at 40°C. The mobile phase consisted of 65:35 acetonitrile/water (v/v) at a flow rate of 0.75 mL min^−1^; the injection volume was 20 µL^−1^. The drug was detected at 230 nm, and the retention time was 5.8 min. The calibration curve was linear over a range of 0.05–50 µg mL^−1^.

### Effects of Formulations on Skin Barrier Function

The effects of the applied formulations on the skin barrier properties were evaluated by measuring transepidermal water loss (TEWL) using an AquaFlux AF 200 instrument (Biox Systems Ltd, UK) at 30–36% relative air humidity and temperature between 24–26 °C. The skin was mounted in Franz cells as described above for the permeation study. First, baseline TEWL before sample application was recorded: the upper part of the diffusion cell was carefully removed, and the TEWL probe was placed on the holder for 80–100 s until a stable value was obtained. The formulations were applied to the skin in the same manner as above; controls were left untreated. After 24 h, the formulations were removed as above, and the skin surface was left exposed to air for 2 h to prevent interference with TEWL. Then, TEWL was measured at predetermined time points up to 10 h after the formulations had been removed.

### Data Analysis

Three or more groups were compared using one‐way ANOVA with Tukey's multiple comparisons test; *p* < 0.05 was considered statistically significant (GraphPad Prism version 9.0.0, GraphPad Software, USA). The data are presented as the means ± standard deviation (SD); the number of replicates (n) is specified in each figure.

## Conflict of Interest

The authors declare no conflict of interest.

## Supporting information

Supporting Information

Supplemental Video 1

## Data Availability

The data that support the findings of this study are available in the supplementary material of this article.
