# Supplementary material for: Topical Drug Delivery of Concentrated Cabazitaxel in an α‐Tocopherol and DMSO Solution
Source: Adv Sci (Weinh). 2023 Aug 9;10(29):2302658. doi: 10.1002/advs.202302658 (PMC10582425; doi:10.1002/advs.202302658)
Supplement: Supplementary file 1 — Supporting Information [file ADVS-10-2302658-s002.pdf]

ADVANCED SCIENCE
Open Access

Supporting Information

for *Adv. Sci.*, DOI 10.1002/advs.202302658

Topical Drug Delivery of Concentrated Cabazitaxel in an α-Tocopherol and DMSO Solution

Boyang Sun, Georgios Paraskevopoulos, Jiwei Min, Robert Rossdeutcher, Sanjana Ghosh, Breandan Quinn, Meng-Hsuan Lin, Debanjan Sarkar, Dinesh Sukumaran, Yuefei Wang, Kateřina Vávrová*, Jonathan F. Lovell* and Yumiao Zhang**

Supporting Information

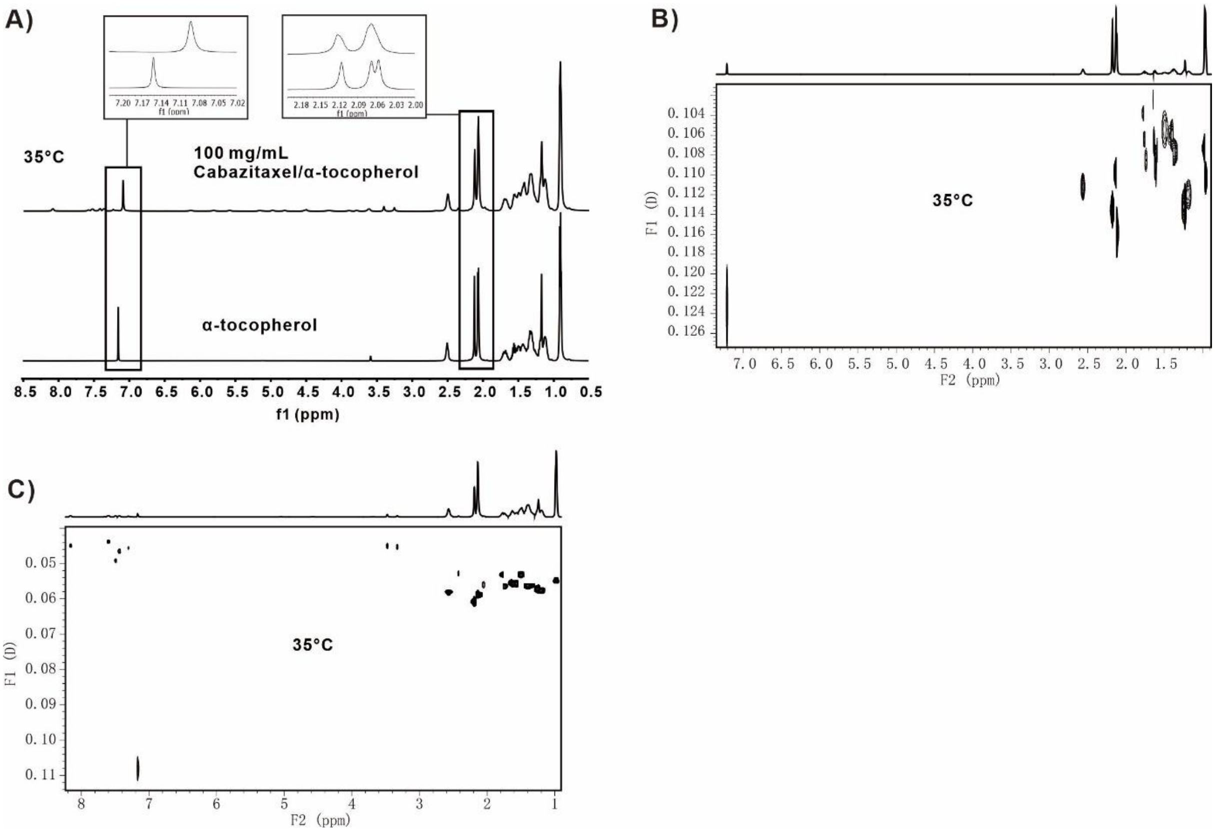

Figure S1. 2-D DOSY Nuclear Magnetic Resonance analysis. A) Stacked 1H-NMR spectra of α-tocopherol (bottom) and 100 mg/mL CTX/ α-tocopherol (top) in 30% DMSO-d_6 at 35°C. **B)** 2D-DOSY spectra of α-tocopherol in 30% DMSO-d_6 (500 MHz, 35°C). **C)** 2D-DOSY spectra of 100 mg/mL CTX/ α-tocopherol in 30% DMSO-d_6 (500 MHz, 35°C).

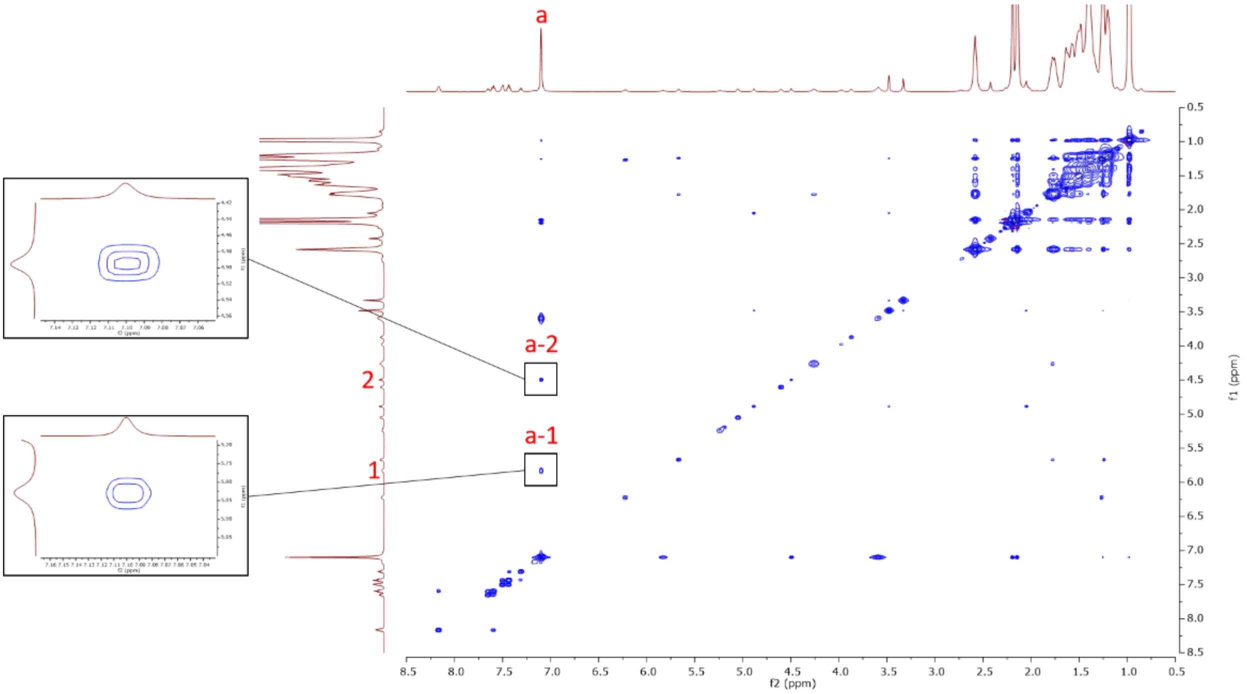

Figure S2. ^{1}H-^{1}H NOESY of 100 mg/mL CTX/ α-tocopherol in 30% DMSO-d_6 (500 MHz, 45°C, 150 ms mixing time)

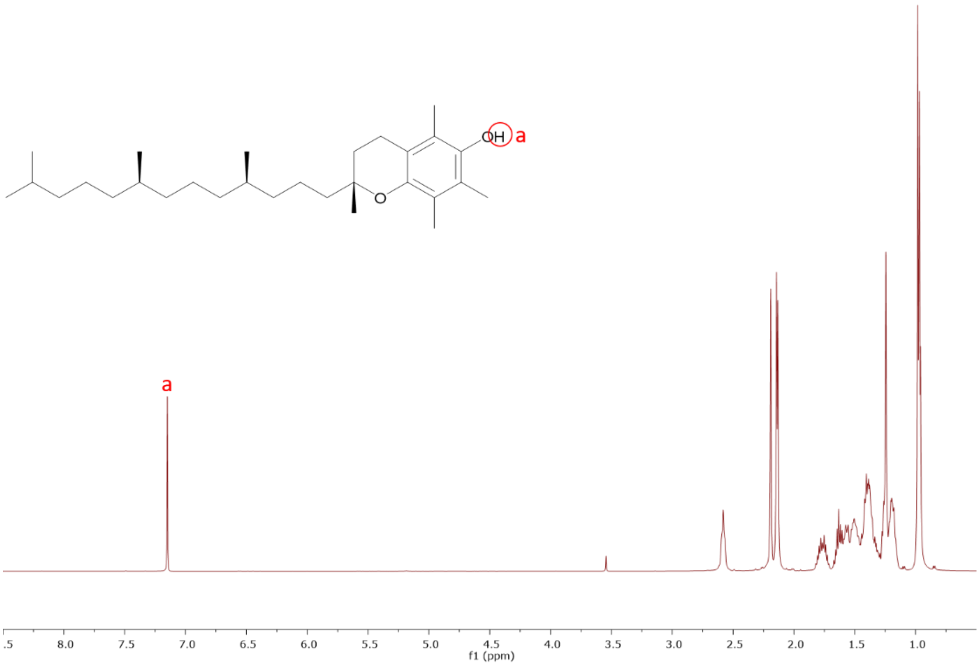

Figure S3. ^{1}H-NMR spectrum of α-tocopherol in 30% DMSO-d_6 (500 MHz, 45°C).

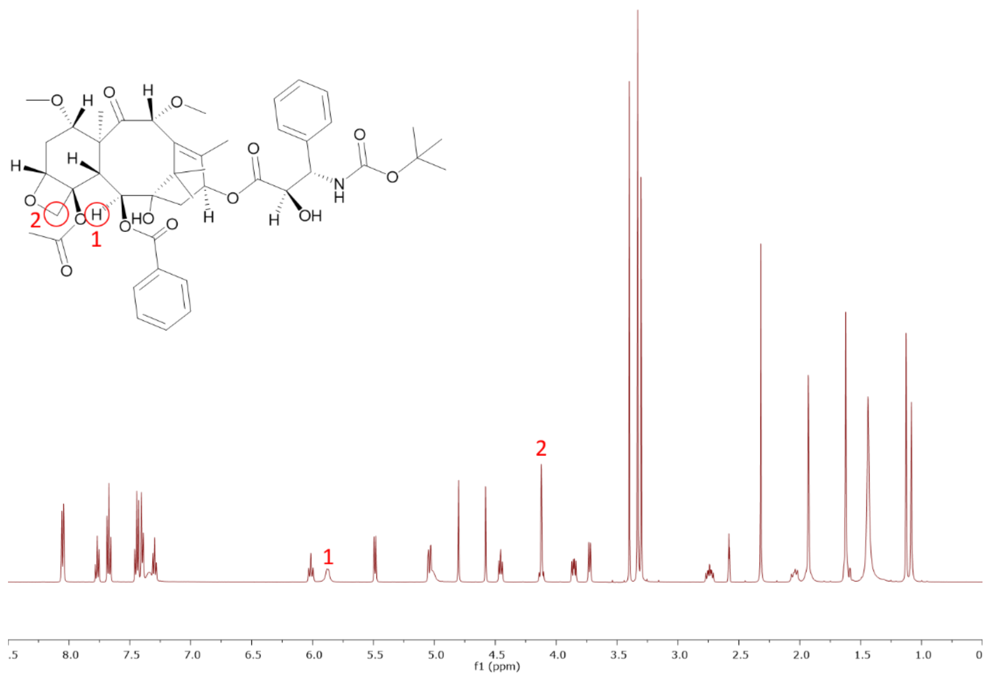

Figure S4. ^{1}H-NMR spectrum of CTX in DMSO-d_6 (70 mg/mL, 500 MHz, 45°C).

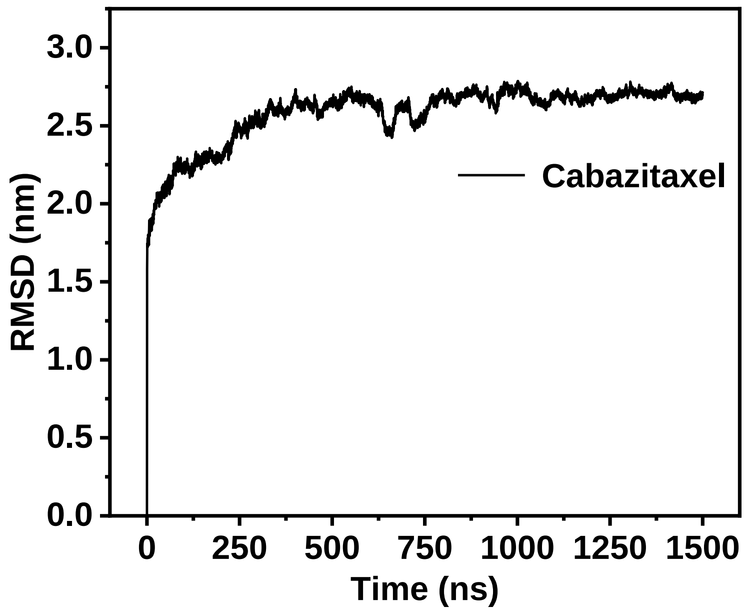

Figure S5. The RMSD of the system during MD simulation.

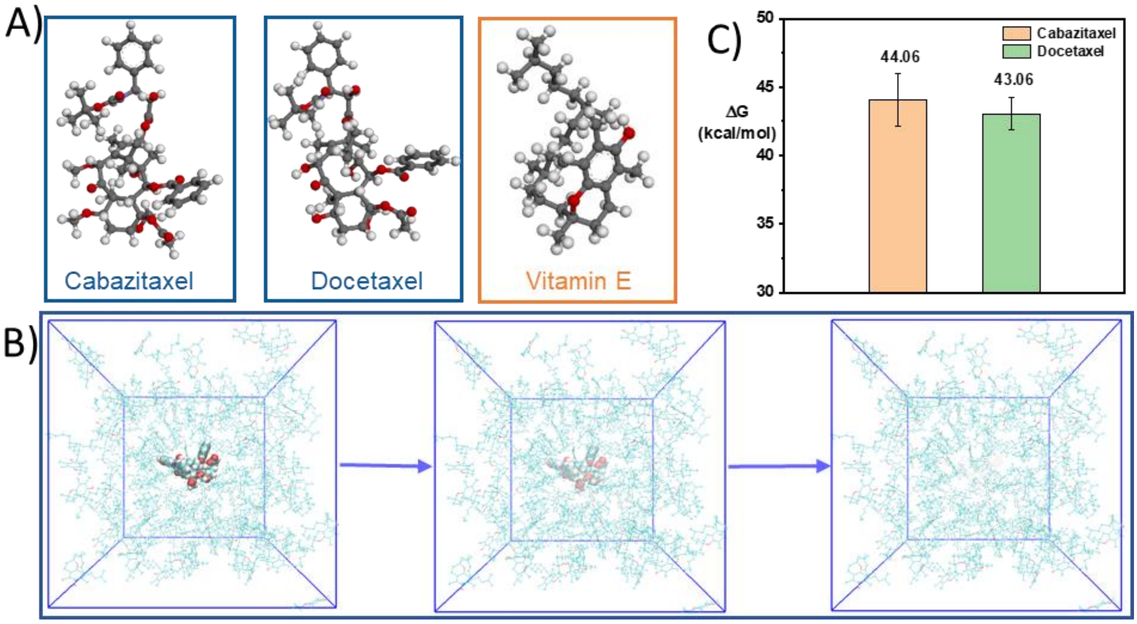

Figure S6. A) Molecular structure of CTX, DTX, and the α-tocopherol. **B)** Linear decoupling of van der Waals interactions between drug molecules and α-tocopherol, where the gradual disappearance of the drug molecule represented the gradual disappearance of the van der Waals interaction between the drug molecule and the solvent molecules. **C)** The free energy change calculated by Gromacs for the two drug molecules with Vitamin E, respectively.

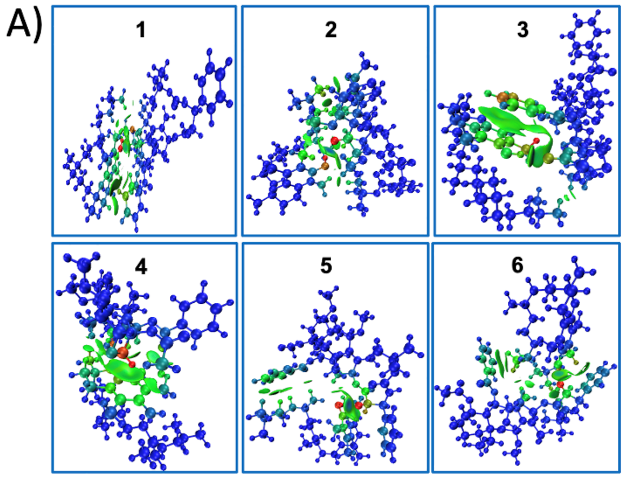

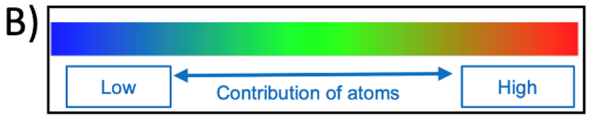

C)

Energy Decomposition based on molecular force field					
	Electrostatic	Repulsion	Dispersion	Total (kJ/mol)	Total (kcal/mol)
1	-23.6	88.2	-141.7	-77.1	-18.4
2	-19.8	56.7	-122.6	-85.7	-20.5
3	-22.0	67.5	-123.0	-77.6	-18.5
4	-19.1	69.5	-128.3	-77.9	-18.6
5	-25.7	81.3	-130.8	-75.1	-18.0
6	-24.5	61.3	-125.0	-88.2	-21.1

Figure S7. A) The contribution of individual atoms to the weak interactions in each configuration, the strength of which is indicated by the colorimetric card in **B),** from which it could be seen that the main weak interactions are π-π stacking between benzene rings and p-π stacking between benzene rings and alkyl chains. **C)** The results of the energy

decomposition in the molecular force field, indicate the dominance of van der Waals interactions.

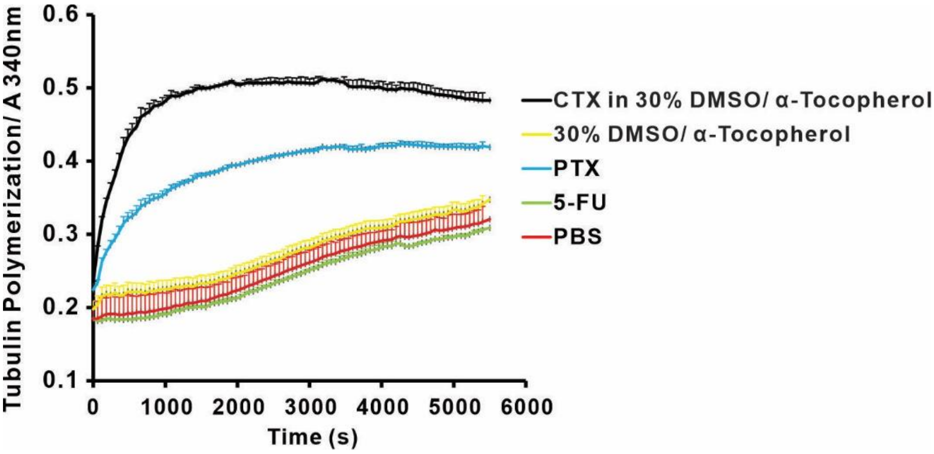

Figure S8. Standard tubulin polymerization reaction rate after treatment by various formulations at 10 μM drug concentration. The datapoints shown represent the average of three replicates of experiments.

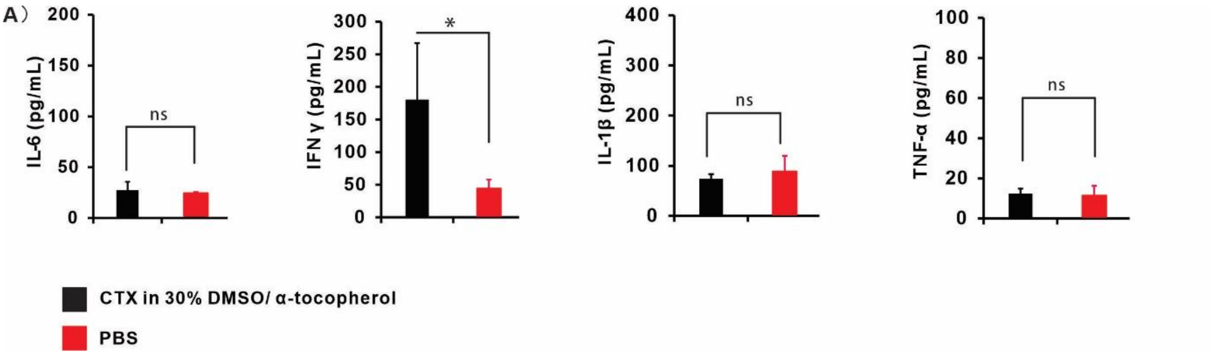

Figure S9. A) Serum levels of inflammatory cytokines after of CD-1 mice were topically administered 20 μL of 75 mg/mL CTX in 30% DMSO/ α-tocopherol on day 1 and sacrificed on day 3 (n=3). Statistically significant differences were analysed by one-way ANOVA in Prism 6.
